# Wnt signaling in regulation of biological functions of the nurse cell harboring *Trichinella* spp.

**DOI:** 10.1186/s13071-016-1770-4

**Published:** 2016-09-02

**Authors:** Magdalena Dabrowska, Marek Skoneczny, Zbigniew Zielinski, Wojciech Rode

**Affiliations:** 1 Laboratory of Comparative Enzymology, Department of Biochemistry, Nencki Institute of Experimental Biology, Polish Academy of Sciences, 3 Pasteur St., Warsaw, 02-093 Poland; 2 Department of Genetics, Institute of Biochemistry and Biophysics, Polish Academy of Sciences, 5A Pawinskiego St., Warsaw, 02-106 Poland

**Keywords:** *Trichinella* spp., Nurse cell, Wnt signaling, Growth arrest, Inflammatory phenotype, AP-1 transcription factor

## Abstract

**Background:**

The nurse cell (NC) constitutes in mammalian skeletal muscles a confined intracellular niche to support the metabolic needs of muscle larvae of *Trichinella* spp. encapsulating species. The main biological functions of NC were identified as hypermitogenic growth arrest and pro-inflammatory phenotype, both inferred to depend on AP-1 (activator protein 1) transcription factor. Since those functions, as well as AP-1 activity, are known to be regulated among other pathways, also by Wnt (Wingless-Type of Mouse Mammary Tumor Virus Integration Site) signaling, transcription profiling of molecules participating in Wnt signaling cascades in NC, was performed.

**Methods:**

Wnt signaling-involved gene expression level was measured by quantitative RT-PCR approach with the use of Qiagen RT^2^ Profiler PCR Arrays and complemented by that obtained by searching microarray data sets characterizing NC transcriptome.

**Results:**

The genes involved in inhibition of canonical Wnt/β-catenin signaling cascade as well as leading to β-catenin degradation were found expressed in NC at high level, indicating inhibition of this cascade activity. High expression in NC of genes transmitting the signal of Wnt non-canonical signaling cascades leading to activation of AP-1 transcription factor, points to predominant role of non-canonical Wnt signaling in a long term maintenance of NC biological functions.

**Conclusions:**

Canonical Wnt/β-catenin signaling cascade is postulated to play a role at the early stages of NC formation when muscle regeneration process is triggered. Following mis-differentiation of infected myofiber and setting of NC functional specificity, are inferred to be controlled among other pathways, by Wnt non-canonical signaling cascades.

**Electronic supplementary material:**

The online version of this article (doi:10.1186/s13071-016-1770-4) contains supplementary material, which is available to authorized users.

## Background

The nurse cell (NC) constitutes an intracellular niche for the muscle larvae of parasitic nematode *Trichinella* spp. Its basic morphological structure, called cyst, is formed within mammalian striated muscles 20–28 days post-oral infection [1, 2]. Larva penetration into the muscles induces degeneration of infected myofiber, followed by its fusion with muscle satellite cells and commencement of regeneration process. However, eventually mis-differentiation takes place and part of the infected myofiber transforms into a non-muscular structure, the NC fulfilling larva metabolic requirements. NC-larva complex confined within a collagen capsule and surrounded by circulatory rete is stably maintained throughout the life span of the host [1]. NC is characterized by hypertrophy and 4 N DNA content [3, 4]. Based on transcription profiling NC growth arrest stage was identified as being of G_1_-like type accompanied by cellular senescence [5]. NC was also found to display antigen presentation capability and pro-inflammatory secretory phenotype [6].

Wnt signaling pathway plays an important role in morphogenesis and postnatal stem cell fate determination [7, 8]. Inhibition of canonical Wnt/β-catenin signaling is required for cell lineage differentiation but the cascade, if recapitulated in mature differentiated cellular systems, is associated with onset of various diseases, including neurodegeneration and malignancies [9–11]. A role in cellular senescence and aging-associated disorders have been ascribed to various Wnt ligands [12–14]. Physiological responses to Wnt signaling are elicited by diverse cellular functions: cell survival, proliferation, apoptosis, differentiation, cell movement and immunological activities [15]. Wnt growth factors bind to transmembrane Frizzled (Fzd) receptors, belonging to G Protein-Coupled Receptor (GPCR) family [9]. The signal is subsequently transduced via three distinct routes: the canonical Wnt/β-catenin and two non-canonical Wnt/PCP (Planar Cell Polarity) and Wnt/Ca^2+^, signaling cascades [15, 16]. Particular Wnt ligand-Fzd receptor interactions are tissue- and process-specific. It is emphasized for Wnt signal transduction that various combinations of ligand-receptor complexes, as well as many regulatory loops and cross-talks, also with other signaling pathways, ultimately lead to a cell-specific type of response [17, 18]. Despite such a diversity, specifically Wnt 4, Wnt 5A and Wnt 11 ligands are considered to activate Wnt non-canonical cascades [18, 19]. Of note, Wnt 5A upregulation was demonstrated to occur in stimulated antigen-presenting cells, i.e. dendritic cells and macrophages [20]. In the case of canonical Wnt signaling route transcription of effector genes is activated by β-catenin transcription activation complex, and in the case of non-canonical Wnt signaling route, by AP-1 transcription factor [15].

As far as skeletal muscles are concerned, Wnt signaling is involved in myogenesis and muscle regeneration. Canonical Wnt/β-catenin signaling mediated by Wnt 1 and Wnt 7A ligands was shown to induce early myogenesis in mice [21]. Wnt 3A, Wnt 5A/5B and Wnt 7A/7B ligands signaling is considered critical for muscle regeneration, with myoblast differentiation and myotube fusion assumed to be affected [8]. Yet transient β-catenin activation, accompanying this process, is also viewed rather as a vestige from embryonic lineage, crucial for myogenesis but requiring inhibition for muscle regeneration to proceed [22].

As a cellular system, NC originates from muscle cells suspended during regeneration. Immunological activities with signaling pathways culminating at AP-1 transcription factor activation, were identified as its prominent biological functions [6]. Those characteristics should apparently be controlled by Wnt signaling. Additionally, Wnt 2 ligand was found in general analysis of NC transcriptome to be highly upregulated, in comparison to myoblastic cell line [5]. Therefore, the present scrutinized analysis was undertaken, of expression level of factors involved in Wnt signaling in NC, performed with the use of PCR arrays and supported by the search of microarray data sets [5]. The results point to a putative essential role of Wnt factors in setting of NC phenotype.

## Methods

### NC isolation

**Fig. 1:**
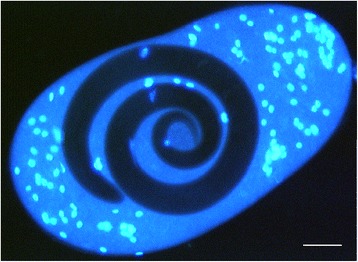
NC-*Trichinella spiralis* larva complex. The nuclei were visualized with Hoechst 33342 dye (Lonza). The image was taken with Nikon Optiphot-Z fluorescence microscope. *Scale-bar*: 100 μm

Trichinellosis in BALB/c mice, infected with *Trichinella spiralis* H2 human isolate, was exploited as previously described [23]. NCs were isolated from mice carrying 6 month-old infections by sequential muscle digestion, as earlier presented [5]. NC in a typical preparation is shown in Fig. 1.

### RT^2^ Profiler PCR Arrays

Qiagen kits were used at all steps. Total RNA was isolated with the use of RNaesy Mini kit, according to manufacturer’s instruction, with implementation of the RNase-Free DNase digestion step. RNA integrity was confirmed using Agilent 2100 BioAnalyzer. RT^2^ First Strand kit was used for reverse transcription. RT^2^ SYBR Green/ROX qPCR Master Mix was used for quantitative PCR on Qiagen RT^2^ Profiler PCR Array of Mouse Wnt Signaling Pathway. The run was performed on 7500 Sequence Detection System (Applied Biosystems), including all control reactions recommended by the arrays’ manufacturer. Target gene expression level was calculated according to Qiagen RT^2^ Profiler PCR Array handbook, applying the comparative threshold cycle (C_T_) method, with glyceraldehyde-3-phosphate dehydrogenase (GAPDH) used as a reference gene. It is given as 2exp-ΔC_T_ (± average deviation for *n* = 2), where ΔC_T_ is C_T_ (target gene)-C_T_ (GAPDH). The genes included into RT^2^ Profiler PCR Array whose threshold cycle fell above 35th cycle were excluded from data presentation.

### Microarray data sets searching

In order to complement performed herein transcription profiling of Wnt signaling factors in NC, the previously obtained competitive microarray data sets [5], were searched for identifiers included in Qiagen RT^2^ Profiler PCR Array, as well as the identifiers not included in the PCR array but otherwise related to Wnt signaling. In the aforementioned competitive microarray analysis, the transcriptomes of C2C12 myoblasts and C2C12 myotubes served as referral systems to the NC transcriptome. In order to eliminate biological differences among NC preparations, four different preparations of NCs isolated from mice carrying 5 to 12 month-infections were exploited for competitive microarray analysis. Only the identifiers with differential gene expression level ≥ 2 accompanied by a *P*-value ≤ 0.05, were considered signaling pathway-eligible. Fold change in gene expression level in NC, in relation to C2C12 myoblasts or myotubes, was calculated form log_2_ratio value and is provided as the average of quadruplicates, accompanied by the *P*-values calculated by Student’s one-sample *t*-test. All parameters of statistical analysis, including log_2_ratio ± standard deviation (SD) as well as the *t*-values, are shown in Additional file 1: Table S1.

## Results and discussion

### Characteristics of NC formation process

The NC is a non-muscular structure originating from a few types of cells. During encapsulation of the larva lasting up to 28 days post-infection, the nuclei, mitochondria and basophilic cytoplasm (i.e. staining with haematoxylin in haematoxylin and eosin (H&E) staining protocol), of infected myofiber, degenerate with the signs of apoptosis and autocrine signaling by tumor necrosis factor α [24, 25]. Inhibition of transforming growth factor β signaling by c-Ski repressor was also shown to accompany this process [26]. Muscle satellite cells, fusing with the infected degenerating myofiber, become the main source of nuclei, mitochondria and eosinophilic cytoplasm (i.e. staining with eosin in H&E staining procedure), in the completely established NC at 3-month-old infection. Some nuclei of NC become hypertrophied at this stage, and infiltrating lymphocytes were also identified entrapped in the NC cytoplasm [27]. It should be noted that during NC formation two various kinds of cytoplasm, basophilic and eosinophilic, are separated by plasma membrane and the whole process in independent on p53 suppressor gene [2, 27, 28]. Analysis of NC transcriptome during the process of intracellular transformation (i.e. 23rd day post-infection), indicated activation of survival mechanism mediated by insulin-like growth factor 1 which may lead to induction of AP-1 transcription factor [29, 30]. Wnt 8A and 5B ligand expression was also found upregulated at this stage of NC development, as analyzed in the whole infected *vs* uninfected muscle tissue [29]. Wnt canonical signaling cascade inhibitory factor Dickkopf homolog 4 (DKK4 gene) [31], was also found upregulated in those settings [29]. These findings indicate that already at the stage of larva encapsulation Wnt signaling-involved factors shape NC functional specificity towards non-canonical Wnt signaling and AP-1 factor activation, serving to determine survival and immunological properties.

### Inhibitory factors of canonical Wnt/β-catenin signaling cascade are expressed in fully established NC

**Fig. 2:**
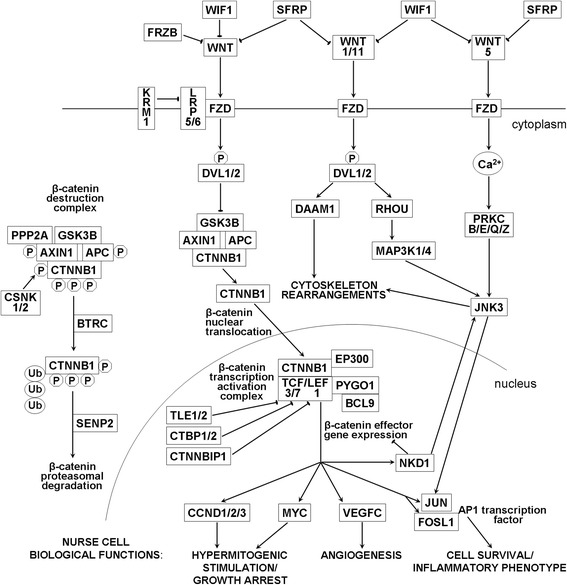
Summary of interactions involved in Wnt signaling cascades inferred to regulate NC biological functions. Only the molecules whose expression was detected in NC by RT^2^ Profiler PCR Arrays and/or microarrays, are marked. Wnt signaling consensus pathway was complied from Wnt signaling pathway available at www.qiagen.com/pl/shop/genes-and-pathways/pathway-details/?pwid=474, Ingenuity Pathway Analysis software (www.ingenuity.com/products.ipa) and references [9, 15]. Sharp arrows indicate activatory interactions and blunt arrows indicate inhibitory interactions. Descriptions of molecule names are given in Table 1

A network of molecules participating in Wnt signaling, whose expression was detected in NC, is depicted in Fig. 2. Gene description and gene expression levels are shown in Table 1. In cells unstimulated by Wnt ligands, central molecule of this cascade, β-catenin (encoded by CTNNB1 gene), is known to remain in the cytoplasm in a phosphorylated form complexed with GSK3B and scaffolding factors APC and AXIN1 [32]. Apart from GSK3B, also casein kinases, represented in NC by CSNK1A1, CSNK1D and CSNK2A1, are known to phosphorylate β-catenin. Additionally, bound with protein phosphatases (PPP2CA, PPP2R1A and PPP2R5D subunits are expressed in NC), β-catenin is ubiqutinated in the presence of BTRC and driven for proteasomal degradation [32]. SENP2 peptidase is also known to participate in downregulation of β-catenin level [33, 34]. Assuming autocrine stimulation to occur, the Wnt/β-catenin signaling cascade can be activated in NC by Wnt 1, Wnt 2/2B, Wnt 3/3A, Wnt 6, Wnt 9A and Wnt 16. Upon stimulation of FZD receptors by Wnt ligands activated Dsh1/2 proteins (encoded by DVL1/2 genes), lead to inhibition of β-catenin phosphorylation. Unphosphorylated β-catenin translocates to the nucleus where it forms a transcription activation complex with TCF/LEF factors, additionally activated by EP300 and BCL9/PYGO1 complex [32]. FZD receptors 1 through 8, coreceptors LRP5/6, as well as DVL1/2, TCF3/7 and LEF1 genes, are expressed in NC, though TCF7 and LEF1 are down regulated in relation to C2C12 cellular systems. Numerous molecules known to inhibit β-catenin transcription activation complex, including TLE1/2, CTBP1/2, CTNNBIP1, as well as an effector, and simultaneously an inhibitor, of this cascade, NKD1 gene product [35], are expressed in NC. NKD1 expression is also very highly upregulated in relation to C2C12 myoblasts/myotubes. Of note, NKD1 is known to switch Wnt signaling from canonical to non-canonical Wnt/PCP cascade [36]. Apart from apparent inhibition of canonical Wnt/β-catenin signaling cascade in NC by intracellular factors, this cascade may also be inhibited at the plasma membrane level. KRM1 (alias KREMEN1), WIF1, FRZB (alias SFRP3) and SFRP1/2/4 gene products, expressed in NC, are known to inhibit Wnt signaling via interaction with LRP5/6 coreceptors (KRM1), binding to Wnt ligands (WIF1 and FRZB) or interaction with both Wnt ligands and Fzd receptors (SFRP1/2/4 factors) [37, 38]. Importance of an inhibitory route of canonical Wnt/β-catenin signaling in NC at the plasma membrane level is further stressed by very high upregulation of FRZB and high upregulation of WIF1 expression in NC, in relation to C2C12 myoblasts/myotubes. Of note is that Wnt 1 inducible signaling pathway protein 1 WISP1, known to display anti-apoptotic activity [39], is expressed in NC at the level significantly lower than in C2C12 cellular systems. Effector genes of canonical Wnt/β-catenin signaling cascade expressed in NC include factors involved in regulation of cell fate and inflammation: cyclins D, c-Myc, Fra1 (encoded by FOSL gene) and c-Jun, as well as angiogenic factor VEGFC [web.stanford.edu/group/nusselab/cgi-bin/wnt/target_genes].

**Table 1:** Expression in NC of molecules involved in Wnt signaling pathway. Gene expression level determined by RT^2^ Profiler PCR Arrays is given as 2^−∆CT^, and by competitive microarray approach as a fold change, increase or decrease (↓) in relation to C2C12 myoblasts and myotubes. Data analysis was performed as described under Methods section

GenBank accession number	Gene symbol	Description	Gene expression 2^−∆CT^ (± AD, *n* = 2)	Fold change (*P*-value) in gene expression level in NC *vs* C2C12 Myoblasts/myotubes
NM_007462	APC	Adenomatous polyposis coli	2.92 ± 0.40	
NM_009733	AXIN1	Axin1	0.14 ± 0.06	
NM_029933	BCL9	B-cell CLL/lymphoma 9	0.16 ± 0.03	
NM_009771	BTRC	Beta-transducin repeat containing protein	0.63 ± 0.04	
NM_023465	CTNNBIP1	Catenin beta interacting protein 1	1.59 ± 0.01	/2.1 (0.00)
NM_007631	CCND1	Cyclin D1	4.40 ± 0.62	/9.5 (0.00)*
NM_009829	CCND2	Cyclin D2	19.33 ± 1.41	5.1 (0.00)*/7.8 (0.00)*
NM_007632	CCND3	Cyclin D3	2.37 ± 0.64	2.1 (0.01)/↓1.9 (0.01)
NM_146087	CSNK1A1	Casein kinase 1, alpha 1	3.85 ± 1.01	/↓2.1 (0.00)
NM_139059	CSNK1D	Casein kinase 1, delta	7.36 ± 1.33	
NM_007788	CSNK2A1	Casein kinase 2, alpha 1 polypeptide	9.55 ± 0.80	↓2.9 (0.00)/↓3.4 (0.00)
NM_013502	CTBP1	C-terminal binding protein 1	0.38 ± 0.12	
NM_009980	CTBP2	C-terminal binding protein 2	5.26 ± 0.17	/2.2 (0.01)
NM_007614	CTNNB1	Catenin (cadherin associated protein), beta 1	2.03 ± 0.40	
NM_172464	DAAM1	Dishevelled associated activator of morphogenesis 1	5.13 ± 0.47	
NM_010091	DVL1	Dishevelled 1, dsh homolog (Drosophila)	0.48 ± 0.13	/↓2.2 (0.00)
NM_007888	DVL2	Dishevelled 2, dsh homolog (Drosophila)	0.09 ± 0.04	
NM_177821	EP300	E1A binding protein p300	0.22 ± 0.06	
NM_010234	FOS	v-FOS murine viral oncogene homolog		116.7 (0.00)*/27.2 (0.00)*
NM_008036	FOSB	FBJ murine viral oncogene homolog		6.0 (0.00)*/5.5 (0.00)*
NM_010235	FOSL1	Fos-like antigen 1	1.36 ± 0.13	↓3.1 (0.00)/2.1 (0.01)
NM_011356	FRZB	Frizzled-related protein	8.25 ± 2.13	21.9 (0.00)/14.8 (0.00)
NM_021457	FZD1	Frizzled homolog 1 (Drosophila)	1.36 ± 0.46	3.6 (0.00)/
NM_020510	FZD2	Frizzled homolog 2 (Drosophila)	1.20 ± 0.77	
NM_021458	FZD3	Frizzled homolog 3 (Drosophila)	0.39 ± 0.04	
NM_008055	FZD4	Frizzled homolog 4 (Drosophila)	0.35 ± 0.00	3.6 (0.01)/
NM_022721	FZD5	Frizzled homolog 5 (Drosophila)	1.97 ± 0.26	
NM_008056	FZD6	Frizzled homolog 6 (Drosophila)	0.22 ± 0.11	
NM_008057	FZD7	Frizzled homolog 7 (Drosophila)	0.07 ± 0.04	
NM_008058	FZD8	Frizzled homolog 8 (Drosophila)	0.02 ± 0.01	3.4 (0.00)/2.4 (0.03)
NM_019827	GSK3B	Glycogen synthase kinase 3 beta	0.75 ± 0.02	
NM_010591	JUN	Jun oncogene	7.68 ± 2.99	1.9 (0.00)/
NM_010592	JUND	Jun-D proto-oncogene		2.5 (0.02)*/2.1 (0.02)*
NM_032396	KREMEN1	Kringle containing transmembrane protein 1	8.56 ± 0.19	
NM_010703	LEF1	Lymphoid enhancer binding factor 1	0.01 ± 0.003	↓3.7 (0.00)/↓2.4 (0.04)
NM_008513	LRP5	Low density lipoprotein receptor-related protein 5	1.43 ± 0.63	3.4 (0.00)/3.9 (0.00)
NM_008514	LRP6	Low density lipoprotein receptor-related protein 6	1.44 ± 0.09	
NM_011945	MAP3K1	MEKK1, MAP kinase kinase kinase 1		4.9 (0.00)*/6.7 (0.00)*
NM_011948	MAP3K4	MEKK4, MAP kinase kinase kinase 4		2.1 (0.00)*/2.0 (0.00)
NM_009158	MAPK10	JNK3, Jun-N terminal kinase		8.8 (0.00)*/6.8 (0.00)*
NM_010849	MYC	Myelocytomatosis oncogene	0.88 ± 0.14	↓4.7 (0.00)*/↓2.9 (0.01)*
NM_027280	NKD1	Naked cuticle 1 homolog (Drosophila)	0.37 ± 0.04	42.3 (0.00)/27.8 (0.00)
NM_008702	NLK	Nemo-like kinase	0.38 ± 0.01	
NM_019411	PPP2CA	Protein phosphatase 2 (formerly 2A), catalytic subunit, alpha isoform	15.79 ± 0.81	
NM_016891	PPP2R1A	Protein phosphatase 2 (formerly 2A), regulatory subunit A (PR 65), alpha isoform	10.09 ± 0.04	
NM_009358	PPP2R5D	Protein phosphatase 2, regulatory subunit B (B56), delta isoform	0.75 ± 0.14	
NM_008855	PRKCB1	Protein kinase C, beta 1		10.0 (0.01)*/6.7 (0.00)*
AK017901	PRKCE	Protein kinase C, epsilon		4.3 (0.01)*/4.4 (0.00)*
NM_008859	PRKCQ	Protein kinase C, theta		6.3 (0.00)*/4.6 (0.01)*
NM_008860	PRKCZ	Protein kinase C, zeta		14.6 (0.00)*/10.6 (0.00)*
NM_028116	PYGO1	Pygopus 1	0.38 ± 0.01	4.6 (0.00)/3.0 (0.01)
NM_133955	RHOU	Ras homolog gene family, member U	0.51 ± 0.16	/2.3 (0.00)
NM_029457	SENP2	SUMO/sentrin specific peptidase 2	1.88 ± 0.05	
NM_013834	SFRP1	Secreted frizzled-related protein 1	0.09 ± 0.008	
NM_009144	SFRP2	Secreted frizzled-related protein 2	0.02 ± 0.012	/↓5.4 (0.00)
NM_016687	SFRP4	Secreted frizzled-related protein 4	0.01 ± 0.003	
NM_009332	TCF3	Transcription factor 7-like 1 (T-cell specific, HMG box)	1.31 ± 0.10	2.1 (0.02)/2.6 (0.00)
NM_009331	TCF7	Transcription factor 7, T-cell specific	0.46 ± 0.13	↓3.2 (0.00)/↓2.3 (0.01)
NM_011599	TLE1	Transducin-like enhancer of split 1	1.10 ± 0.27	
NM_019725	TLE2	Transducin-like enhancer of split 2	0.005 ± 0.0022	
NM_009506	VEGFC	Vascular endothelial growth factor C		5.3 (0.00)*/5.9 (0.00)*
NM_011915	WIF1	Wnt inhibitory factor 1	0.15 ± 0.02	6.4 (0.00)/2.5 (0.00)
NM_018865	WISP1	WNT1 inducible signaling pathway protein 1	2.22 ± 0.69	↓4.9 (0.00)/↓6.1 (0.00)
NM_021279	WNT1	Wingless-related MMTV integration site 1	0.003 ± 0.0011	
NM_009519	WNT11	Wingless-related MMTV integration site 11	1.89 ± 0.22	6.5 (0.00)/4.1 (0.00)
NM_053116	WNT16	Wingless-related MMTV integration site 16	0.14 ± 0.01	5.6 (0.01)/3.3 (0.00)
NM_023653	WNT2	Wingless-related MMTV integration site 2	0.008 ± 0.0029	28.2 (0.00)*/21.9 (0.00)*
NM_009520	WNT2B	Wingless related MMTV integration site 2b	0.03 ± 0.001	
NM_009521	WNT3	Wingless-related MMTV integration site 3	0.002 ± 0.0013	3.3 (0.01)/2.4 (0.00)
NM_009522	WNT3A	Wingless-related MMTV integration site 3A	0.004 ± 0.0022	
NM_009523	WNT4	Wingless-related MMTV integration site 4	0.009 ± 0.0062	↓2.9 (0.00)/
NM_009524	WNT5A	Wingless-related MMTV integration site 5A	0.03 ± 0.011	4.8 (0.01)/3.0 (0.02)
NM_009525	WNT5B	Wingless-related MMTV integration site 5B	5.89 ± 2.90	9.9 (0.00)/7.7 (0.00)
NM_009526	WNT6	Wingless-related MMTV integration site 6	0.03 ± 0.008	↓2.8 (0.01)/
NM_139298	WNT9A	Wingless-type MMTV integration site 9A	0.01 ± 0.005	2.5 (0.00)/↓2.1 (0.00)

*Asterisks mark gene expression level, determined by microarray approach and reported previously in the context of other signaling pathway analyses [5, 6]

It is thus inferred that dominant expression in NC of molecules involved in β-catenin degradation as well as inhibition of canonical Wnt signal transduction and β-catenin-dependent transcription, indicate that even though could be operating, Wnt/β-catenin signaling cascade is inhibited. Expression of the cascade effector genes may have resulted from Wnt/β-catenin-activated transcription at the earlier stages of NC formation, but in fully established NC this regulation seems to be attributed rather to other signaling pathways.

### Effector factors of non-canonical Wnt/PCP and Wnt/Ca^2+^ signaling cascades are expressed in fully established NC

A network of molecules participating in non-canonical Wnt signaling cascades, whose expression was detected in NC, is schematically depicted in Fig. 2, with gene descriptions and expression level values provided in Table 1. Non-canonical Wnt signaling was shown in various cellular systems to be stimulated by Wnt 1/11 and Wnt 5A ligands [15, 40, 41]. Wnt 11 and Wnt 5B are expressed in NC at the highest level among other Wnt ligands. Their expression, as well as the expression of Wnt 5A, is also upregulated in relation to C2C12 myoblasts/myotubes. Similar to the canonical cascade, activation of Wnt/PCP cascade occurs via phosphorylation of Dsh proteins [16]. In NC, the signal can be transduced downstream by DAAM1 factor and RHOU-MAP3K1/4-JNK3 axis, to induce cytoskeleton rearrangements. JNK3 can also be activated in NC via Ca^2+^ and protein kinase C axis, known to be activated also by classical GPCRs. JNK3 phosphorylates c-Jun and JunD which then dimerise with one of the Fos proteins to form transcription factor AP-1, known to display prosurvival and proinflammatory action, as well as to inhibit myogenesis [16, 42–47]. Expression of c-Jun, JunD, Fra-1 (encoded by FOSL1 gene), FosB and Fos is found in NC, with Fos being the most highly upregulated gene in relation to C2C12 cellular systems. Thus expression in NC of Wnt 11, Wnt 5A/5B ligands, as well as JNK3 and Jun/Fos factors, indicate importance of AP-1 factor in maintenance of NC biological functions mediated by non-canonical Wnt signaling cascades. One of the effector genes of Wnt/Ca^2+^ signaling cascade, expressed in NC, is Nemo-like kinase (NLK, Table 1). As NLK is known to suppress β-catenin-dependent transcription [48], its expression in NC further points to inhibition of canonical Wnt signaling cascade.

It is inferred from the study performed that canonical, as well as non-canonical cascades operate in NC at the various stages of its formation. Expression in the fully established NC of Wnt ligands responsible for activation of canonical Wnt signaling cascade, the latter known to accompany induction of muscle regeneration [8, 22], may reflect the vestiges from those stages of NC formation when muscle regeneration was triggered. Eventually the cascade inhibition prevails. It is also possible that expression in fully established NC of the cascade inhibiting factors, including a feedback inhibitor NKD1, is indicative of execution of a tight control of the remaining activity of canonical Wnt signaling. It can be hypothesized that at the time point of larva penetration Wnt autocrine signaling may be responsible for β-catenin-dependent induction of infected myofiber regeneration. As no differentiation ultimately occurs, probably due to influence of EGF (epidermal growth factor)/FGF (fibroblast growth factor)/PDGF (platelet-derived growth factor)- induced proliferative stimulation [5], Wnt 5A/5B- and Wnt 11-activated non-canonical signaling cascades sustain the activation of AP-1 transcription factor to regulate NC growth arrest and immunological functions [6]. Current analysis was based on putative loops of autocrine signaling operating in NC. Parasite-derived factors and paracrine signaling should also control NC formation and the functioning of NC at fully established stage. Long-term maintenance of NC biological specificity apparently results from a precise orchestration of various cellular signaling events. The nature of the exact factor causing transformation of muscular cells to the parasite-favorable environment, remains to be identified.

## Conclusions

The NC is an intracellular habitat for *Trichinella* spp. muscle larvae. Assuming autocrine signaling by Wnt ligands to occur during a long-term existence of the NC-*Trichinella* muscle larva complex, the canonical Wnt signaling cascade is inferred to be inhibited, but the non-canonical Wnt/PCP and Wnt/Ca^2+^ cascades are postulated to lead to maintenance of AP-1 transcription factor activation and execution of NC biological functions.

## Additional file

Additional file 1: Table S1. The parameters of statistical analysis of competitive expression microarray data showing gene expression level in NC related to either C2C12 myoblasts or myotubes. Only the genes referred to in Table 1 and Fig. 2 of the main body of the publication, are shown. (DOC 70 kb)

## Abbreviations

AP-1: activator protein 1
EGF: epidermal growth factor
FGF: fibroblast growth factor
GAPDH: glyceraldehyde-3-phosphate dehydrogenase
GPCR: G protein–coupled receptor
H&E: haematoxylin and eosin
NC: nurse cell
PCP: planar cell polarity
PDGF: platelet-derived growth factor
Wnt: Wingless-type of mouse mammary tumor virus integration site
