# Supplementary material for: Wnt signaling in regulation of biological functions of the nurse cell harboring Trichinella spp
Source: Parasit Vectors. 2016 Sep 2;9(1):483. doi: 10.1186/s13071-016-1770-4 (PMC5010673; doi:10.1186/s13071-016-1770-4)
Supplement: Additional file 1: Table S1. — The parameters of statistical analysis of competitive expression microarray data showing gene expression level in NC related to either C2C12 myoblasts or myotubes. Only the genes referred to in Table 1 and Fig. 2 of the main body of the publication, are shown. (DOC 70 kb) [file 13071_2016_1770_MOESM1_ESM.doc]

**Additional file 1: Table 1.** The parameters of statistical analysis of competitive expression microarray data showing gene expression level in NC related to either C2C12 myoblasts or myotubes. Only the genes referred to in Table 1 and Figure 2 of the main body of the publication, are shown

| **GenBank accession number** | **Gene symbol** | **NC *vs* C2C12 myoblasts** | | | **NC *vs* C2C12 myotubes** | | |
| --- | --- | --- | --- | --- | --- | --- | --- |
|  |  | **Average log_2_ratio ± SD, *N* = 4** | ***t*-value** | ***P*-value** | **Average log_2_ratio ± SD, *N* = 4** | ***t*-value** | ***P*-value** |
| NM_023465 | CTNNBIP1 |  |  |  | 1.053 ± 0.173 | 12.140 | 0.001 |
| NM_007631 | CCND1 |  |  |  | 3.241 ± 0.293 | 22.107 | 0.000 |
| NM_009829 | CCND2 | 2.363 ± 0.195 | 24.285 | 0.000 | 2.969 ± 0.180 | 32.947 | 0.000 |
| NM_007632 | CCND3 | 1.083 ± 0.360 | 6.006 | 0.009 | 0.952 ± 0.282 | -6.749 | 0.007 |
| NM_146087 | CSNK1A1 |  |  |  | -1.082 ± 0.153 | -14.160 | 0.001 |
| NM_007788 | CSNK2A1 | -1.555 ± 0.283 | -10.980 | 0.002 | -1.751 ±0.222 | -15.756 | 0.001 |
| NM_009980 | CTBP2 |  |  |  | 1.127 ± 0.311 | 7.248 | 0.005 |
| NM_010091 | DVL1 |  |  |  | -1.154 ± 0.167 | -13.797 | 0.001 |
| NM_010234 | FOS | 6.867 ± 0.434 | 31.624 | 0.000 | 4.768 ± 0.763 | 12.500 | 0.001 |
| NM_008036 | FOSB | 2.589 ± 0.565 | 9.169 | 0.003 | 2.467 ± 0.567 | 8.697 | 0.003 |
| NM_010235 | FOSL1 | -1.620 ± 0.228 | -14.234 | 0.001 | 1.037 ± 0.309 | 6.717 | 0.007 |
| NM_011356 | FRZB | 4.454 ± 0.795 | 11.205 | 0.002 | 3.887 ± 0.528 | 14.718 | 0.001 |
| NM_021457 | FZD1 | 1.863 ± 0.375 | 9.947 | 0.002 |  |  |  |
| NM_008055 | FZD4 | 1.834 ± 0.569 | 6.450 | 0.008 |  |  |  |
| NM_008058 | FZD8 | 1.752 ± 0.418 | 8.394 | 0.004 | 1.270 ± 0.650 | 3.908 | 0.030 |
| NM_010591 | JUN | 0.943 ± 0.120 | 15.652 | 0.001 |  |  |  |
| NM_010592 | JUND | 1.320 ± 0.520 | 5.084 | 0.015 | 1.081 ± 0.423 | 5.113 | 0.015 |
| NM_010703 | LEF1 | -1.867 ± 0.457 | -8.176 | 0.004 | -1.256 ± 0.715 | -3.511 | 0.039 |
| NM_008513 | LRP5 | 1.756 ± 0.404 | 8.673 | 0.003 | 1.948 ± 0.351 | 11.100 | 0.002 |
| NM_011945 | MAP3K1 | 2.296 ± 0.380 | 12.078 | 0.001 | 2.744 ± 0.334 | 16.432 | 0.000 |
| NM_011948 | MAP3K4 | 1.070 ± 0.073 | 29.458 | 0.000 | 0.983 ±0.135 | 14.569 | 0.001 |
| NM_009158 | MAPK10 | 3.137 ± 0.194 | 32.431 | 0.000 | 2.766 ± 0.095 | 58.232 | 0.000 |
| NM_010849 | MYC | -2.219 ± 0.381 | -11.660 | 0.001 | -1.553 ± 0.568 | -5.480 | 0.012 |
| NM_027280 | NKD1 | 5.403 ± 0.564 | 19.161 | 0.000 | 4.799 ± 0.529 | 18.147 | 0.000 |
| NM_008855 | PRKCB1 | 3.328 ± 1.275 | 5.221 | 0.014 | 2.753 ± 0.492 | 11.192 | 0.002 |
| AK017901 | PRKCE | 2.090 ± 0.541 | 7.720 | 0.005 | 2.129 ± 0.478 | 8.908 | 0.003 |
| NM_008859 | PRKCQ | 2.662 ± 0.226 | 23.550 | 0.002 | 2.211 ± 0.627 | 7.052 | 0.006 |
| NM_008860 | PRKCZ | 3.872 ± 0.215 | 36.052 | 0.000 | 3.410 ± 0.328 | 20.782 | 0.000 |
| NM_028116 | PYGO1 | 2.206 ± 0.303 | 14.537 | 0.001 | 1.613 ± 0.560 | 5.765 | 0.010 |
| NM_133955 | RHOU |  |  |  | 1.172 ± 0.192 | 12.219 | 0.001 |
| NM_009144 | SFRP2 |  |  |  | -2.424 ± 0.473 | -10.248 | 0.002 |
| NM_009332 | TCF3 | 1.055 ±0.489 | 4.312 | 0.023 | 1.386 ± 0.288 | 9.627 | 0.002 |
| NM_009331 | TCF7 | -1.697 ± 0.340 | -9.989 | 0.002 | -1.171 ± 0.430 | -5.452 | 0.012 |
| NM_009506 | VEGFC | 2.407 ± 0.267 | 18.028 | 0.000 | 2.566 ± 0.232 | 22.135 | 0.000 |
| NM_011915 | WIF1 | 2.669 ± 0.266 | 20.067 | 0.000 | 1.329 ± 0.280 | 9.507 | 0.002 |
| NM_018865 | WISP1 | -2.284 ± 0.488 | -9.361 | 0.003 | -2.603 ± 0.422 | -12.349 | 0.001 |
| NM_009519 | WNT11 | 2.706 ± 0.266 | 20.342 | 0.000 | 2.052 ± 0.047 | 86.667 | 0.000 |
| NM_053116 | WNT16 | 2.493 ± 0.782 | 6.375 | 0.008 | 1.733 ± 0.375 | 9.230 | 0.003 |
| NM_023653 | WNT2 | 4.818 ± 0.558 | 17.256 | 0.000 | 4.454 ± 0.350 | 25.454 | 0.000 |
| NM_009521 | WNT3 | 1.709 ± 0.491 | 6.956 | 0.006 | 1.247 ± 0.127 | 19.563 | 0.000 |
| NM_009523 | WNT4 | -1.542 ± 0.297 | -10.399 | 0.002 |  |  |  |
| NM_009524 | WNT5A | 2.262 ± 0.599 | 7.548 | 0.005 | 1.573 ± 0.698 | 4.507 | 0.020 |
| NM_009525 | WNT5B | 3.310 ±0.643 | 10.300 | 0.002 | 2.948 ± 0.198 | 29.744 | 0.002 |
| NM_009526 | WNT6 | -1.497 ± 0.452 | -6.627 | 0.007 |  |  |  |
| NM_139298 | WNT9A | 1.327 ± 0.211 | 12.575 | 0.001 | -1.096 ± 0.203 | -10.793 | 0.002 |
